# Effects of Rumen-Protected Methionine on Meat Quality, Fatty Acid Composition, Volatile Flavor Compounds and Transcriptomics of *Longissimus lumborum* of Yak (*Bos grunniens*)

**DOI:** 10.3390/foods14122102

**Published:** 2025-06-15

**Authors:** Xia Wu, Zizhen Zuo, Jiajia Li, Jianhui Fu, Jincheng Zhong, Hui Wang, Haitao Shi, Yanling Huang, Haibo Wang

**Affiliations:** 1 Key Laboratory of Animal Science of National Ethnic Affairs Commission of China, Southwest Minzu University, Chengdu 610041, China; wufengxia0512@163.com (X.W.); ljiajia2025@126.com (J.L.); zhongjincheng518@126.com (J.Z.); wanghui892321@swun.edu.cn (H.W.); shihaitao010@163.com (H.S.); dmxsnow@yahoo.com.cn (Y.H.); 2 Key Laboratory of Qinghai-Tibetan Plateau Animal Genetic Resource Reservation and Utilization, Southwest Minzu University, Chengdu 610041, China; cairangji120@163.com (Z.Z.); fujianhui2022@126.com (J.F.); 3 Sichuan Zoige Alpine Wetland Ecosystem National Observation and Research Station, Southwest Minzu University, Chengdu 610041, China

**Keywords:** rumen-protected methionine, *Longissimus lumborum*, meat quality, fatty acid, volatile flavor compounds, transcriptomics

## Abstract

Yak (*Bos grunniens*) meat is popular with a unique flavor and high nutritional value. This study investigated the effects of dietary supplementation with rumen-protected methionine (RPM) on meat quality, fatty acid composition, volatile flavor compounds, and transcriptomics of *Longissimus lumborum* of yak. Twenty-four male Maiwa yaks were selected and assigned to four groups: basal diet (CON), or supplementation of 5 g/d (RPM5), 10 g/d (RPM10), and 15 g/d (RPM15) RPM. The dose-dependent effects of RPM levels were evaluated through linear or quadratic trend analysis. The results showed that diet supplementation with RPM increased the intramuscular fat contents, improved composition of volatile flavor compounds and the ratio of monounsaturated fatty acids to saturated fatty acids. Compared to the CON group, there were 36, 84 and 23 up-regulated genes, and 85, 94 and 70 down-regulated genes in the RPM5, RPM10 and RPM15 groups, respectively. Gene ontology enrichment analysis revealed significant differentially expressed genes enrichment in biological processes, cellular components, and molecular functions across RPM5, RPM10, and RPM15 groups compared to the CON. KEGG pathway analysis revealed 99, 169, and 104 enriched pathways in RPM5, RPM10, and RPM15 groups, respectively. In summary, the addition of RPM to diets may provide new ideas and methods to improve meat quality of yaks.

## 1. Introduction

The unique ecological adaptability of yaks makes them an important livestock resource in plateau areas. Their meat, milk, hide, and wool not only enrich the food and economic sources of local residents but also promote the development of the regional economy and the improvement of farmers’ living standards [1]. Compared to beef cattle, yak meat is higher in protein, lower in fat, and richer in minerals and unsaturated fatty acids (UFA) [2]. In recent years, with the increasing demand of consumers for high-quality meat, the market for yak meat has expanded rapidly. Under traditional grazing, yaks face significant weight loss and high mortality during the long cold season from October to May [3]. The survival strategy of yaks shows obvious seasonal adaptability [4]. This greatly restricts the development of the yak industry and the improvement of meat quality. However, due to high breeding costs and low production efficiency, the economic benefits of yak breeding still need to be improved. Therefore, it is crucial to take scientific, ecological, and standardized measures to enhance the productivity and meat quality of yaks.

Methionine (Met), one of the first two limiting amino acids for ruminants, plays crucial roles in regulating protein synthesis, lipid metabolism, and DNA methylation, serving as a precursor for bioactive compounds and participating in redox reactions [5,6]. Additionally, Met can effectively enhance growth efficiency, reproductive performance and slaughter performance in livestock [7,8]. Low-protein diets supplementation with Met has been shown to increase milk production and milk protein levels in dairy cows [9]. Studies have shown that supplementing with 0.06% rumen-protected methionine and 0.08% guanidinoacetic acid in Tan lambs can significantly improve growth performance and meat quality [10]. Dietary supplementation with Met can improve carcass quality, influence the expression of amino acid transporter protein genes, and promote intestinal amino acid absorption in finishing crossbred steers [11]. The inclusion of rumen-protected methionine (RPM) in low-protein diets can modulate fat deposition by regulating lipogenesis and lipolysis in the liver and muscle [12]. Furthermore, Met promotes muscle growth by downregulating the mRNA of the muscle growth inhibitor *MSTN* and upregulating the mRNA expression of myogenic growth factors such as *Myf5* and *MEF2B* [13].

The above studies demonstrate that Met enhances protein and fat deposition in ruminants and modulates the expression of related pathways and cytokines that influence myocyte growth. Our previous research has shown that RPM acts as a multifunctional modulator in ruminant nutrition, effectively optimizing carbohydrate metabolism, modulating the structural and functional dynamics of rumen microbiota, and boost growth performance [14,15]. At the same time, studies have shown that volatile compounds in meat are the source of aroma, and volatile flavor compounds make important contributions to the formation of meat flavor [16]. Among them, sulfur-containing compounds are considered particularly important. The key reaction that produces sulfur-containing compounds is the Maillard reaction between sulfur-containing amino acids and reducing sugars, which generates a variety of sulfur-containing meat flavor compounds. Methionine is the only sulfur-containing essential amino acid and may be related to the formation of these flavor compounds [17]. However, the effects of RPM on yak meat quality and the underlying mechanism are still unclear. Therefore, this study was designed to investigate the effects of RPM on the meat quality, fatty acid composition, volatile flavor compounds, and transcriptomics in *Longissimus lumborum* of yaks.

## 2. Materials and Methods

### 2.1. Feeding and Management

Feeding and slaughtering tests were completed at Shengyuan Yak Husbandry Co., Ltd. in Xiaojin County, Aba Prefecture, Sichuan Province (102°59′ E, 30°35′ N, altitude at 2500 m). All procedures of this study have been approved by the Animal Care Committee of Southwest Minzu University (Protocol number: SMU 202106010). In this experiment, 24 healthy Maiwa male yaks (age 4.0 ± 0.3 years, body weight: 252.79 ± 15.95 kg) were selected and randomly divided into four groups: basal diet (CON), basal diet supplemented with 5 g/d RPM (RPM5), basal diet supplemented with 10 g/d RPM (RPM10) and basal diet supplemented with 15 g/d RPM (RPM15). All experimental yaks were fed individually, with six replicate pens per treatment group and one yak per pen. The animals were fed twice daily (at 7:30 a.m and 3:30 p.m.) with ad libitum total mixed ration (TMR), and water was provided evenly after feeding. The experimental animals were housed in a 12 m × 8 m (96 m^2^) structure with corrugated metal roofing (3.5 m ridge height), accommodating 24 yaks at a density of 4 m^2^ per yak. The flooring consisted of a compacted crushed stone base. The RPM (MetaSmart, Adisseo Inc., Antony, France) was individually sprinkled on the surface of the total mixed ration (TMR) for each yak. Based on our previous research, for every 10 g of dry matter (DM) consumed, yaks ingest 2.22 g of methionine (Met) [14]. The proportions and chemical composition of the basal diet are listed in Table 1. A ten-day acclimatization trial was conducted first, followed by a seventy-day continuous trial, all experimental yaks were expelled of worm with a dose of 0.2 mg/kg body weight ivermectin (Qinghai Animal Pharmaceutical Co., Ltd., Hainan Tibetan Autonomous Prefecture, Xining, China) via subcutaneous injection at day 3 of the acclimatization trial.

### 2.2. Sample Collection and Indicator Determination

The slaughtering process strictly follows the Beef Grading Standards (NY/T676-2003). Approximately 5 g of *Longissimus lumborum* samples were collected immediately, placed in microcentrifuge tubes, and flash-frozen in liquid nitrogen to preserve biomolecular integrity for further analysis. After the carcass was aged at 4 °C for 24 h, a sample of the *Longissimus lumborum* about 10 cm thick was collected from each yak, then the pH, Brightness (L*), redness (a*), yellowness (b*), dripping loss, cooking loss, Warner-Bratzler Shear Force (WBSF) were detected. Other samples were placed in self-sealing bags, then embedded in dry ice, transported back to the laboratory, and stored in a −20 °C freezer for dry matter, crude protein and crude fat analysis.

#### 2.2.1. Meat Quality and Nutrient Content

(1) The pH value of the *Longissimus lumborum* was measured 24 h post-mortem using a pH meter (Testo 205, testo AG, Schwarzwald, Germany). The instrument was calibrated at pH 4.0 and 7.0, with the probe inserted at the 12th rib interface following the triple-point penetration protocol [18]. Three measurements per sample were averaged. (2) *L* *, *a* * and *b* * were measured after 24 h of aging using a chroma meter (CR-400/410, Konica Minolta, Tokyo, Japan) with 8 mm and 50 mm apertures. Values were recorded from three randomized locations on the muscle surface. The instrument was pre-calibrated with a white reference tile [19]. (3) Three 2 × 3 × 5 cm *Longissimus lumborum* samples parallel to the muscle fiber orientation were hung up for 24 h in a 4 °C sealed environment to calculate the drip loss. (4) Cooking loss was determined by the following process: weighing ≈ 80 g rectangular meat samples (m_1_) Vacuum-sealing samples in heat-resistant bags, heating in a water bath at 80 °C until core temperature reached 70 °C, cooling to room temperature, blotting surface moisture, and re-weighing samples (m_2_); cooking loss (%) = [(m_1_ − m_2_)/m_1_] × 100. (5) WBSF was measured using a texture analyzer (Instron 4411, Instron Corporation, Norwood, MA, USA) with a 100 N load cell and crosshead speed of 200 mm/min. Cylindrical cores (1.27 cm diameter) were sheared perpendicular to muscle fiber orientation. Three replicates per sample were averaged according to AMSA guidelines [20]. (6) The dry matter content was determined using a freeze dryer (FC-40, Atlascopco, Rock Hill, SC, USA): weighing fresh samples (m_3_), slicing into ≈5 mm sections, pre-freezing at −80 °C for ≥4 h, freeze-drying for 24 h at (−50 ± 5) °C under (10 ± 5) Pa vacuum, and re-weighing (m_4_); moisture (%) = [(m_3_−m_4_)/m_3_] × 100. (7) Crude fat content was determined via weighing freeze-dried samples (m_5_), extracting (FatExtractor E-500, Buchi, Flawil, Switzerland) in petroleum ether (110 °C) for 240 min using an automated Soxhlet extractor, air-drying in fume hood, then oven-drying at 65 °C for 4 h, Final weighing (m_6_); crude fat (%) = [(m_5_−m_6_)/m_5_] × 100. (8) Crude protein content of freeze-dried *Longissimus lumborum* samples was quantified using the Dumas Nitrogen Analyzer (NDA702, VELP, Usmate Velate, Italy) [21].

#### 2.2.2. Fatty Acid Composition

Fatty acids were determined following the procedures described by Song et al. [22]. About 0.3 g of the meat sample was put into a test tube, then 1 mL of 10 mol/L potassium hydroxide and 8 mL methanol were added to the tube. The tube was heated in a water bath at 50 °C for 30 min. Subsequently, 0.9 mL of 12 mol/L sulfuric acid was added, and the tube was heated in a water bath at 80 °C for 1 h. The sample was extracted by adding 3 mL of n-hexane, and then centrifuged to obtain the supernatant, which was dehydrated by anhydrous sodium sulfate and passed through a 0.22 μm filter membrane. It was detected using gas chromatography (7890B, Agilent Technologies, Santa Clara, CA, USA). Gas chromatography conditions: Separation was performed on an HP-88 capillary column (100 m × 250 μm × 0.2 μm) with a split-mode injector (9:1 ratio, 240 °C, constant flow 1 mL/min). Detection used FID (240 °C) with gas flows of 450 mL/min (air), 40 mL/min (H_2_), and 30 mL/min (makeup gas). The oven program started at 100 °C (hold 3 min), ramped at 10 °C/min to 175 °C (hold 10 min), then 5 °C/min to 210 °C (hold 12 min), and finally 5 °C/min to 230 °C (hold 10 min). Compounds were quantified based on relative peak area percentages.

### 2.3. Determination of Volatile Flavor Compounds

Referring to the method of Wang et al. [23], 5 g of minced meat sample was taken in a headspace vial, equilibrated at 80 °C for 10 min, and then headspace adsorbed using SPME fiber at the same temperature for 30 min. Gas chromatography-mass spectrometry (Agilent Technologies 7890B, USA) desorption conditions: desorption was carried out at 230 °C in the inlet port for 2 min using a TG-WAXMS column with helium as the carrier gas, and the temperature was programmed to be increased (held at 40 °C for 3 min, 4 °C/min to 210 °C for 5 min). The mass spectrometer interface temperature was 210 °C, with a mass scan range of 40 to 500 *m*/*z*.

ROAV analyzed with reference to Zhang et al. [24]: The ROAV for each volatile flavor compound is calculated using the following formula: ROAV_i_ = 100 × (C_i_/T_i_)/(C_stan_/T_stan_). Here, C_i_ is the relative content (%) of the volatile flavor compound; T_i_ is the olfactory threshold (μg/kg) of the volatile compound; C_stan_ is the proportion of abundance (%) of the main volatile compound in the sample that contributes most to flavor; T_stan_ is the odor detection threshold for that compound (μg/kg).

### 2.4. RNA Quantification, Qualification, Quality Control and qPCR Validation

Twenty-four frozen tissue samples (stored at −80 °C), with six biological replicates per group, were processed by grinding appropriate amounts of tissue in liquid nitrogen. Total RNA was then extracted using the TRIzol method (Invitrogen, Carlsbad, CA, USA) and then subjected to stringent quality control assessment by an Agilent Bioanalyzer 2100 system (RNA 6000 Nano kit, Agilent Technologies, Santa Clara, CA, USA) to verify RNA integrity and purity. Subsequently, double-stranded cDNA was synthesized using M-MuLV reverse transcriptase under optimized reaction conditions. The library was purified using AMPure XP beads (AMPure XP beads, Beckman Coulter, Danvers, MA, USA), amplified via high-fidelity PCR, clustered on an Illumina cBot system (TruSeq PE Cluster Kit v3, Illumina, San Diego, CA, USA), and ultimately sequenced on the HiSeq 2500 platform.

Raw sequencing data (FASTQ format) were initially processed using in-house Perl scripts to remove reads containing adapter sequences, poly-N stretches, or low-quality bases, thereby generating high-quality clean data. Based on the clean data, clusterProfiler R package was used for differential gene GO and KEGG pathway enrichment analysis and combined with a GSEA tool to complete gene set enrichment. SNP detection was realized by GATK (v4.1.1.0).

Total RNA was extracted from *Longissimus lumborum* muscle samples (50 mg) using 1 mL of Trizol and two magnetic beads. The extracted RNA was reverse transcribed to cDNA using the PrimeScript RT Enzyme Mix I kit (Takara, Osaka, Japan), and relative expression was analyzed using the SYBlPremix Ex Taq kit (Takara). The transcript levels of all expressed genes were standardized by *GAPDH*. All primers were synthesized by Tsingke Biotechnology Co., (Beijing, China), with detailed sequences provided in Supplementary Table S1.

### 2.5. Statistical Analysis

Statistical analysis was performed using SPSS 20.0, with graphical representations generated in Origin 2020. The dose-dependent effects of RPM levels were evaluated through linear or quadratic trend analysis; Duncan’s multiple range tests was used to test for significant differences between groups. Group differences in qPCR gene expression data were analyzed by an independent *t*-test. Experimental results are presented as mean and pooled standard errors of the mean (SEM); differences were statistically significant when *p* < 0.05.

## 3. Result

### 3.1. Effects of RPM on Meat Quality and Nutrients of Yak

As shown in Table 2, the slaughter performance, meat color, pH, cooking loss, dripping loss, shear force, DM and protein of *Longissimus lumborum* of yak were not affected by the supplementation of RPM to diet. The intramuscular fat content of *Longissimus lumborum* increased with RPM supplementation (linear, *p* < 0.05); compared to the RPM5 and RPM10 groups, the RPM15 group has the highest intramuscular fat content.

### 3.2. Effects of RPM on Fatty Acid Profiles in Longissimus lumborum of Yak

As shown in Table 3, the dietary supplementation of RPM5 and RPM10 led to a decrease in the percentage of C16:0 and SFA (*p* < 0.05). The addition of RPM in the diet increased the ratio of MUFA to SFA, with the RPM10 group showing a significant increase compared to the RPM5 and RPM15 groups (*p* < 0.05).

### 3.3. Effects of RPM on Volatile Flavor Compounds in Longissimus lumborum of Yak

As shown in Table 4, supplementation with RPM5 and RPM15 significantly decreased the content of 1-octanal (*p* < 0.05). Concurrently, the RPM10 group showed a significant reduction in 2-octanone content, whereas the RPM15 group exhibited an opposite trend. Among hydrocarbon compounds, RPM5 and RPM10 supplementation significantly reduced the content of n-nonadecane (*p* < 0.05). Furthermore, supplementation with RPM5 and RPM10 also significantly decreased n-octane levels, with RPM5 demonstrating a more pronounced reduction compared to RPM10 and RPM15 (*p* < 0.05). In comparison to RPM5 and RPM10, RPM15 supplementation significantly increased the content of hexanol, Butyl carbinol, and 1-octen-3-ol in the alcohol fraction (*p* < 0.05).

As shown in Table 5, the ROAV (Relative Odor Activity Value) analysis revealed that RPM supplementation increased the ROAV values of aldehyde compounds heptanal, lauric aldehyde, 1-octanal, 1-nonanal, and hexanal to above 1. Notably, in the RPM10 group, the ROAV value of 1-nonanal reached 100, indicating its highest contribution to odor perception. Among the alcohol components, hexanol, heptanol, octanol, and 1-octen-3-ol all exhibited ROAV values greater than 1, highlighting their pivotal role in flavor composition. Moreover, in the RPM5 and RPM15 groups, 1-octen-3-ol demonstrated the most significant aromatic contribution, with its ROAV value peaking at 100.

### 3.4. Transcriptomic Data Analysis

#### 3.4.1. Differentially Expressed Gene Analysis

**Figure 1:**
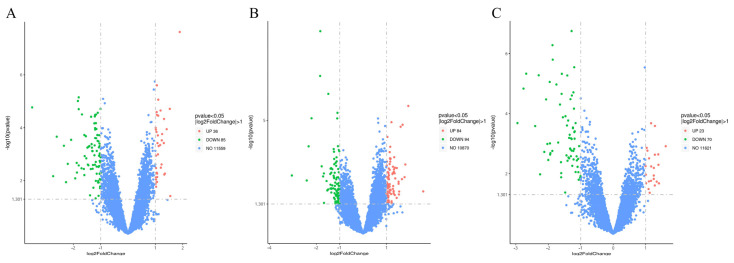
Volcano diagram of differential genes. (**A**) RPM5 vs. CON; (**B)** RPM10 vs. CON; (**C**) RPM15 vs. CON).

The differentially expressed genes (DEGs) in the *Longissimus lumborum* of yaks were analyzed using the DESeq2 software (1.20.0) (*p* value ≤ 0.05, |log_2_FoldChange| ≥ 1.0), as shown in Figure 1. Compared to the CON group, the RPM5 group exhibited 121 DEGs, including 36 significantly upregulated genes and 85 significantly downregulated genes. In the RPM10 group, 178 DEGs were identified, with 84 significantly upregulated and 94 significantly downregulated. The RPM15 group showed 93 DEGs, of which 23 were significantly upregulated and 70 were significantly downregulated.

#### 3.4.2. Differential Gene Ontology Functional Enrichment Analysis

**Figure 2:**
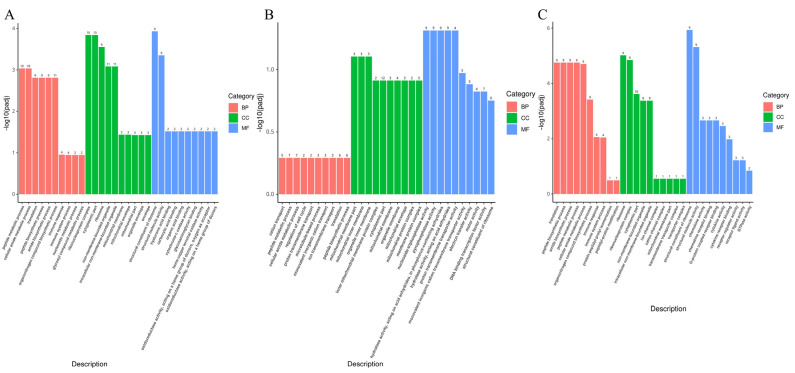
GO enrichment histogram. ((**A**) GO enrichment histogram between RPM5 and CON; (**B**) GO enrichment histogram between RPM10 and CON; (**C**) GO enrichment histogram between RPM15 and CON; BP, Biological Process; CC, Cellular Component; MF, Molecular Function).

As shown in Figure 2, the GO enrichment analysis of differentially expressed genes was performed using the clusterProfiler software (version 3.8.1), which was used to analyze the physiological regulatory mechanisms of DEGs in *Longissimus lumborum* of yaks supplemented with RPM. Compared to the CON group, the RPM5 group showed 37 differential gene annotations for biological process, 25 for cellar components, and 48 for molecular functions. In the RPM10 group, there were 54 differential gene annotations for biological process, 44 for cellular components, and 83 for molecular function. The RPM15 group exhibited 25 differential gene annotations for biological process, 18 for cellular composition, and 31 for molecular function. Biological processes such as peptide metabolism, cytosolic amide metabolism, and translation were significantly enriched in the RPM5 and RPM15 groups. Diet supplemented with RPM significantly enriched cellular components and molecular functions. Specifically, cellular components were significantly enriched in regions such as ribosomes, ribosomal protein complexes, inner membranes of organelles, and inner membranes of mitochondria. Molecular functions were significantly enriched in structural components of ribosomes, structural molecular activity and nucleoside triphosphatase activity.

#### 3.4.3. Differential Gene KEGG Enrichment Analysis

**Figure 3:**
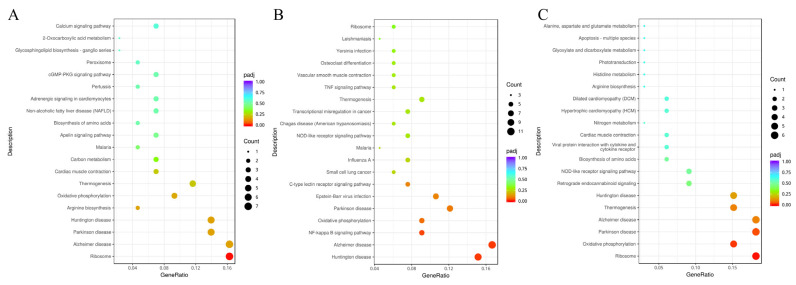
Bubble map of KEGG pathway enrichment. ((**A**) bubble map of KEGG pathway enrichment between RPM5 and CON; (**B**) bubble map of KEGG pathway enrichment between RPM10 and CON; (**C**) bubble map of KEGG pathway enrichment between RPM15 and CON).

As shown in Figure 3, the KEGG pathway analysis of DEGs in yak *Longissimus lumborum* was performed using Gene Set Enrichment Analysis (GSEA). Compared to the CON group, 43 DEGs were enriched in 99 pathways in the RPM5 group, 66 DEGs were enriched in 169 pathways in the RPM10 group, and 33 DEGs were enriched in 104 pathways in the RPM15 group. Significant enrichment was observed in pathways such as ribosome and oxidative phosphorylation.

#### 3.4.4. Validation of Differentially Expressed Genes

**Figure 4:**
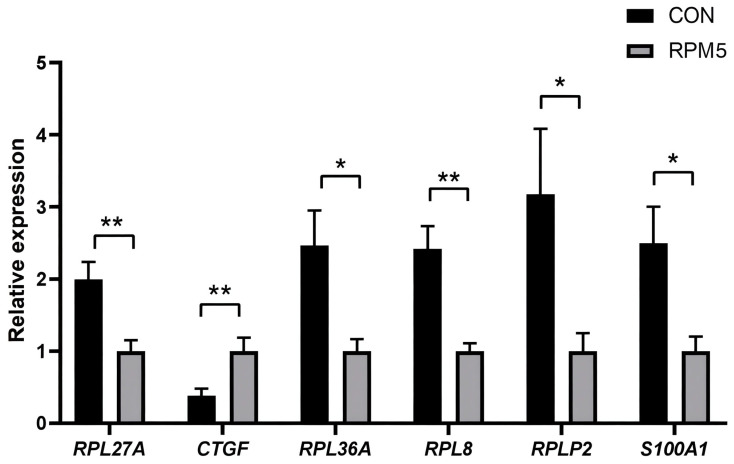
Protein synthesis and animal growth and development related genes expression. * *p* < 0.05, ** *p* < 0.01.

Six DEGs related to protein synthesis and animal growth and development, including ribosome protein L27A (RPL27A), connective tissue growth factor (CTGF), ribosome protein L36A (RPL36A), ribosome protein RPL8 (RPL8), ribosomal stalk protein (RPLP2) and S100 calcium-binding protein A1 (S100A1) were selected to validate the RNA-Seq results using qPCR. Compared with the CON group, the expression of *CTGF* was significantly upregulated (*p* < 0.05), while the expressions of *RPL27A*, *RPL36A*, *RPL8*, *RPLP2* and *S100A1* were significantly downregulated (*p* < 0.05) (Figure 4). Consistent with the transcriptomic data, the expression patterns of these genes were in full agreement with the qPCR results.

## 4. Discussion

Meat quality is an important indicator for measuring the economic efficiency of modern meat products, as it effectively reflects the consumption performance and potential value of meat [25]. Studies have shown that the initial pH value of beef after slaughter is usually between 6 and 7, while the muscle pH value typically decreases to the range of 5.4 to 5.6 due to lactic acid fermentation [26]. In the current study, the pH values of *Longissimus lumborum* of yaks fed different levels of RPM ranged from 5.30 to 5.43, indicating good meat quality. Wu et al. [27] found that when pH is less than 5.79, the degradation of beef major structural proteins, such as titin, nebulin, and filamin is accelerated, which benefits meat tenderness. This study found that the pH values of *Longissimus lumborum* of yaks fed with different levels of RPM were all below 5.79, which can effectively improve the tenderness of the meat. Meat color as a primary visual perception indicator in the consumer sensory evaluation system, and changes in meat color are primarily related to myoglobin and hemoglobin [28]. Studies have shown that under aerobic conditions, the intrinsic reducing systems within muscle tissue significantly reduce metmyoglobin (MetMb) accumulation by converting ferric iron (Fe^3+^) in MetMb to ferrous iron (Fe^2+^). This reduction process triggers the oxygenation cycle of myoglobin: the newly formed deoxymyoglobin (DeoxyMb) rapidly binds with oxygen to generate oxymyoglobin (MbO_2_); consequently, meat color shifts from the brownish hue dominated by MetMb to the bright cherry-red characteristic of MbO_2_, thereby effectively enhancing color stability [29,30]. In the present study, dietary supplementation with different levels of RPM had no effect on meat color or cooking loss of yak *Longissimus lumborum*. Meanwhile, meat tenderness can be measured by shear force. Studies have found that supplementing with rumen-protected methionine can significantly reduce the shear force in Tan lambs, thereby significantly improving the tenderness of the meat [19]. Destefanis et al. [31] defined beef with shear force values of <42.72 N, 42.83–52.63 N and >52.72 N as tender, moderately tender, and tough, respectively. In the present study, the shear force values of the treatments ranged from 42.83 to 52.63 N, indicating that the tenderness of yak meat was classified as moderately tender. Protein and fat contents are directly related to the nutritional value of meat [32]. Intramuscular fat affects the juiciness, tenderness, and flavor of meat [33]. In this study, the intramuscular fat content increased linearly with RPM supplementation, suggesting that the addition of RPM to the diet could effectively improve tenderness of yak meat.

Fatty acids are divided into SFA, MUFA, and PUFA. The main function of SFA is to build body tissues and provide energy; however, excessive intake can lead to elevated blood lipids, particularly serum cholesterol, and increase the risk of cardiovascular diseases, which is detrimental to health [34]. Studies have shown that C12:0, C14:0, and C16:0 are considered to the most harmful fatty acids for the human cardiovascular system [35], with C16:0 potentially increasing blood cholesterol levels [36]. In the present study, dietary supplementation with RPM quadratically decreased the percentages of C16:0 and SFA, indicating that diets supplemented with 5 g and 10 g RPM are beneficial for improving fatty acid composition of yak meat. MUFA has significant benefits for human health by lowering serum total cholesterol levels and delaying atherosclerotic plaque formation, with C18:1 n9c being the most representative fatty acid [37]. In addition, MUFA-enriched dietary interventions not only effectively ameliorate hepatic steatosis in obese rat models but also induce significant remodeling of hepatic phospholipid fatty acid composition profiles [38], and they also help prevent human cardiovascular, atherosclerotic, and thrombotic diseases [39]. In the present study, the most abundant fatty acid in *Longissimus lumborum* of yak was oleic acid, which plays a role in preventing atherosclerosis by lowering blood cholesterol levels [40], suggesting that yak meat has the potential to reduce the risk of cardiovascular diseases. Although dietary supplemented with RPM did not affect the percentage of MUFA, it quadratically increased the ratio of MUFA to SFA, indicating that the RPM optimized fatty acid composition of yak meat. PUFA is closely related to human health, both n-3 and n-6 PUFA can enhance immunity, reduce inflammation, cholesterol, and triglyceride levels, and decrease lipid peroxidation [41,42]. C18:2n6c, a PUFA, is an essential fatty acid that can only be obtained from dietary sources and has beneficial effects on human health, including anti-cancer, anti-inflammatory, and cardiovascular disease preventive effects [43]. In this study, dietary supplementation with RPM tended to linearly and quadratically increase the percentage of C18:2n6c, suggesting that RPM5 and RPM10 improve the fatty acids profiles in *Longissimus lumborum* of yak. When the PUFA/SFA ratio exceeds 0.4, the meat is considered to be of high quality [44]. In this study, the PUFA/SFA ratios in all treatments were higher than 0.4. Additionally, dietary supplementation with RPM tended to quadratically increase the ratio of PUFA to SFA, indicating that *Longissimus lumborum* of the yak was of high quality, and RPM supplementation could further enhance the fatty acid profile of yak meat. Consistent with our results, Zhou et al. [45] demonstrated that dietary supplementation with RPM optimized milk fat composition and milk quality while increasing lactation yield and milk fat content in dairy cows.

Volatile flavor compounds play a significant role in determining the sensory properties of meat [46]. The study results showed that a total of 30 volatile flavor compounds were detected in *Longissimus lumborum* of yaks, including nine aldehydes, seven alcohols, five hydrocarbons, three esters, two ketones, and four other compounds, with the majority consisting of aldehydes, alcohols, hydrocarbons, and esters. The results showed that dietary supplementation with RPM significantly optimized the composition and content of flavor-related compounds in the muscle, thereby enhancing the nutritional value of yak meat. Aldehydes, which are primarily generated through fatty acid oxidation, contribute a characteristic fresh aroma to meat and play a critical role in defining the flavor profile of beef [47]. In this study, dietary supplementation with RPM led to a significant linear increase in the content of Lauric aldehyde among aldehydes as the RPM dosage increased, suggesting that moderate RPM addition in the diet can enhance the content and diversity of aldehydes in *Longissimus lumborum* of yaks. Alcohol compounds, representative of fresh aroma substances, primarily originate from lipid oxidation, spices, or the reduction in aldehydes [48]. The research results showed that dietary RPM supplementation induced significant linearly and quadratically increases in the levels of oct-1-en-3-ol, trans-2-octen-1-ol, and butyl carbinol among alcohols, while also causing a significant quadratic elevation in the content of hexanol. Oct-1-en-3-ol, a predominant volatile flavor compound in alcohols, imparts a mushroom-like aroma and significantly contributes to beef flavor, indicating that RPM supplementation influences beef flavor through oct-1-en-3-ol. Additionally, hexanol, generated via the reduction in hexanal or oxidation of linoleic acid, exhibits a green grassy aroma and plays a modifying role in beef flavor [49]. This implies that RPM supplementation enhances beef flavor by promoting hexanol production.

Volatile compounds with ROAV values greater than 1 are considered major contributors to overall flavor, while those with ROAV values between 0.1 and 1 exhibit modifying effects. The study identified five aldehydes, four alcohols, and one other compound as key contributors to the overall flavor profile. Aldehydes, characterized by intense aromatic properties, directly shape the dominant flavor of beef, geranyl 1-nonanal having floral and fruity notes and lauric aldehyde having citrus and fatty aromas create distinct olfactory characteristics readily perceived by consumers [50]. In this study, dietary RPM supplementation increased the ROAV values of lauric aldehyde and 1-nonanal, with geranyl aldehyde exhibiting the highest odor contribution, indicating that the 10 g RPM supplementation group effectively improved yak beef flavor quality. Among alcohols, hexanol, heptanol, octanol, and 1-octen-3-ol all showed ROAV values exceeding 1, with 1-octen-3-ol demonstrating the highest odor contribution.

*RPL27A*, *CTGF*, *RPL36A*, *RPL8*, *RPLP2*, and *S100A1* were identified as genes associated with the growth and developmental of yaks. *RPL27A* is a ribosomal protein that plays vital roles in ribosomes construction and the regulation of fat formation [51]. The study found that SNPs in the *RPL27A* could enhance the expression of intramuscular fat-related genes, contributing to the superior marbling of Japanese Black Wagyu cattle [52]. *CTGF* is one of the matrix proteins that plays a critical role in promoting skeletal muscle growth and development, accelerating neovascularization, repairing cartilage tissue, promoting mitosis, and stimulating cell proliferation, differentiation and apoptosis [53,54,55]. In the present study, the *CTGF* gene was significantly upregulated with RPM supplementation, consistent with our previous findings that RPM improved the growth performance of yaks [14]. Additionally, the *CTGF* gene has been shown to regulate bone development and chondrogenesis, indirectly reflecting the body shape and growth of livestock [56]. *RPL36A*, a member of the L36AE family, is an important regulatory factor associated with cell cycle regulation and cell proliferation [57]. *RPL8*, belonging to the ribosomal proteins *L2P* family, is involved in protein synthesis in eukaryotic cells [58]. High expression pf *RPL8* in the mammary tissues of lactating dairy cows is associated with SNPs in the promoter region, which also influence milk fat percentage, milk yield, and protein content [59]. *RPLP2* enhances protein stability and is involved in multi-pathway bioprocessing of transmembrane proteins [60]. *S100A1*, a member of the *S100* protein family, regulates fundamental cellular and molecular functions, and maintenance of dynamic nitric oxide homeostasis [61]. Indicating that most of the genes identified through differential transcriptome analysis were related to cell proliferation and differentiation, protein synthesis, fat formation, and bone growth and development.

GO enrichment analysis revealed that protein synthesis in *Longissimus lumborum* of yaks was improved following the addition of RPM to the diet. Compared to the CON group, the DEGs in the RPM5 and RPM15 groups were mainly enriched in biological processes such as translation, polypeptide biosynthesis, and cytosolic amide metabolism, as well as cellular components such as ribonucleoprotein complexes and ribosomes. Ribonucleoprotein complexes are important components of ribosomes and play a key role in the protein translation [62]. Studies have shown that RPM increases the availability of Met in the small intestine, thereby promoting peptide chain synthesis and elongation [63]. During the perinatal period, dietary supplementation with RPM increases hepatic glutathione concentration and contributes to glutathione synthesis in bovine liver [64]. The Met also plays vital roles in regulating energy homeostasis and lipid metabolism, indirectly affecting energy availability during peptide metabolism [65]. In this study, dietary supplementation with RPM may promote peptide chains elongation, enhance the efficiency of polypeptide biosynthesis, and further improve the total amount and quality of proteins in yaks. GO enrichment analysis also revealed that the DEGs in the RPM10 group were enriched in cellular components and molecular functional regions compared to the CON group. Cellular components were primarily enriched in mitochondrial membrane fractions, while molecular functional regions were enriched in nucleoside triphosphatase activity, pyrophosphatase activity, and proton transmembrane transporter protein activity. Mitochondria are the energy factories of the cell, and their membrane components play a critical role in energy conversion and transport. The structural and functional integrity of mitochondrial is essential for meat color stability [66]. Belskie et al. [67] demonstrated that mitochondrial biochemical properties in beef are muscle specific, with the oxygen consumption rate of *Longissimus lumborum* muscle decreasing faster than that of *Longissimus lumborum* muscle RPM may indirectly influence the composition and function of mitochondrial membrane fractions by regulating the activity of mitochondrial membrane transporter proteins and enzymes, optimizing mitochondrial energy metabolism processes, and increasing overall cellular energy levels. This helps maintain the normal structure and function of mitochondria and ensures energy supply [68]. The addition of RPM affected nucleoside triphosphatase activity by modulating energy metabolism processes, as well as the activity of pyrophosphatases and hydrolases, which are involved in the intracellular regulation of various biochemical reactions and metabolic pathways. RPM promotes the synthesis and activity regulation of these enzymes by increasing Met utilization, thereby influencing the metabolic and physiological state of cells. Therefore, dietary supplementation with RPM can improve amino acid utilization in yaks and enhance their productive performance and health status through a complex network of cellular composition, biological processes, and molecular functions.

KEGG enrichment analysis provides a comprehensive understanding of the biological functions of genes at a systems level. In this study, KEGG enrichment analysis showed that dietary supplementation with RPM enriched DEGs mainly in signaling pathways such as oxidative phosphorylation and ribosomes. Key proteins in the oxidative phosphorylation pathway have significant effects on pH, meat color, and relative myoglobin content [69]. Additionally, changes in oxidative phosphorylation of key proteins may lead to metal ion overload during storage, activating endogenous enzymes that hydrolyze muscle structures. These changes in muscle microstructure also affect light reflection, scattering and absorption, resulting in meat color and luster [70]. Ribosome biogenesis serves as a critical process for enhancing protein synthesis and cellular growth. Studies demonstrate that the mTORC1 signaling pathway and the transcription factor c-Myc play central roles in regulating ribosome biogenesis by promoting ribosomal protein synthesis and rRNA transcription [71]. In summary, the addition of RPM to the diet may affect the meat color and protein synthesis of yak beef through pathways such as oxidative phosphorylation and ribosomes.

## 5. Conclusions

Dietary supplementation with RPM could increase intramuscular fat content, effectively improving the compositions of fatty acid and volatile flavor compounds of the *Longissimus lumborum* of yaks. Transcriptome sequencing analysis demonstrated that RPM enhances amino acid utilization and protein synthesis in yaks through enriching pathways such as oxidative phosphorylation and ribosome. Given the increasing demand for high-quality meat, the supplementary feeding amount was 10 g·d^−1^, which is significant implications for the yak farming industry.

## Figures and Tables

**Table 1:** Ingredients and chemical composition of basic diets.

Item	
Ingredients (% DM ^1^)	
Soybean	4.50
Corn meal	15.75
Rapeseed meal	2.25
Soybean meal	7.65
Soybean hull	3.15
Sprayed corn bran	4.50
Corn germ meal	3.60
Molasses	2.25
Corn straw silage	55.0
Premix ^2^	1.35
Chemical composition	
Net energy for gain (MJ/kg)	3.52
Net energy for maintenance (MJ/kg)	5.58
Crude protein (% DM)	13.13
Neutral detergent fiber (% DM)	43.92
Acid detergent fiber (% DM)	15.63
Organic matter (% DM)	90.75

^1^ DM: dry matter. ^2^ Mineral and vitamin premixes contain: vitamin A, 2500 IU; vitamin D, 550 IU; vitamin E, 10 IU; Fe, 40 (mg/kg); Zn, 40 (mg/kg); Cu, 10 (mg/kg); Mn, 40 (mg/kg); I, 0.5 (mg/kg); Co, 0.2 (mg/kg); and Se, (0.2 mg/kg).

**Table 2:** Effect of RPM on slaughter performance and meat quality of yak.

Index	Treatments				SEM	*p*-Value	
	CON	RPM5	RPM10	RPM15		Linear	Quadratic
Final body weight (kg)	306.5	311.3	311.6	314.8	4.124	0.504	0.801
Hot carcass weight (kg)	147.0	150.8	156.7	156.0	3.459	0.298	0.558
Dressing percent (%)	47.86	48.37	50.13	49.40	0.538	0.190	0.366
pH	5.34	5.36	5.41	5.43	0.025	0.162	0.384
L*	34.33	34.46	33.86	35.50	0.288	0.269	0.231
a*	20.14	20.02	19.25	20.67	0.289	0.760	0.407
b*	6.78	6.57	5.99	7.01	0.244	0.930	0.466
Dripping loss (%)	13.26	14.56	13.10	14.07	0.662	0.876	0.982
Cooking loss (%)	29.96	30.58	31.43	30.77	0.570	0.534	0.715
Shear force (N)	45.72	43.73	46.61	45.79	1.207	0.783	0.938
Dry matter (%)	24.80	24.95	24.07	25.01	0.238	0.914	0.717
Fat (%, DM)	12.03	12.07	12.33	13.53	0.266	0.042	0.068
Protein (%, DM)	80.14	80.22	80.61	80.94	0.377	0.426	0.725

**Table 3:** Effects of RPM on fatty acid profiles in *Longissimus lumborum* of yak.

Index	Treatments				SEM	*p*-Value	
	CON	RPM5	RPM10	RPM15		Linear	Quadratic
C14:0	2.08	2.60	1.61	2.42	0.150	0.981	0.767
C14:1	1.40	1.75	1.13	1.24	0.101	0.238	0.427
C15:1	1.83	2.39	2.41	2.44	0.205	0.325	0.515
C16:0	17.83 ^a^	15.70 ^b^	16.54 ^b^	18.70 ^a^	0.466	0.417	0.042
C16:1	4.53	3.17	7.09	2.85	0.482	0.798	0.330
C17:1	0.91	0.81	0.84	0.91	0.036	0.928	0.550
C18:0	12.45	10.08	11.44	12.41	0.493	0.785	0.238
C18:1n9t + C18:1n9c	28.11	28.73	29.02	30.56	0.579	0.142	0.322
C18:2n6c	17.20	18.58	16.70	14.05	0.688	0.063	0.055
C18:3n6	2.40	2.86	2.12	2.33	0.153	0.500	0.740
C20:3n3 + C20:4n6	6.77	9.05	7.43	8.07	0.521	0.631	0.669
C22:2	1.89	1.99	1.25	1.91	0.128	0.568	0.487
SFA	32.36 ^a^	28.38 ^b^	29.59 ^b^	33.53 ^a^	0.822	0.531	0.037
MUFA	36.78	36.85	40.48	38.01	0.546	0.137	0.166
PUFA	28.26	32.47	27.50	26.36	0.923	0.203	0.152
MUFA/SFA	1.14 ^b^	1.30 ^ab^	1.41 ^a^	1.15 ^b^	0.042	0.743	0.030
PUFA/SFA	0.89	1.15	0.97	0.95	0.050	0.346	0.069

SFA, saturated fatty acids; MUFA, monounsaturated fatty acids; PUFA, polyunsaturated fatty acids. Different letters (a, b) marked on the same row of data indicate significant differences (*p*< 0.05).

**Table 4:** Effects of RPM on volatile flavor compounds in *Longissimus lumborum* of yak (%).

Index	Treatments				SEM	*p*-value	
	CON	RPM5	RPM10	RPM15		Linear	Quadratic
Aldehyde							
Benzaldehyde	17.88	18.91	16.06	14.11	1.483	0.296	0.520
3-Methylcrotonaldehyde	0.37	0.40	0.28	0.37	0.014	0.053	0.016
heptanal	1.32	1.48	1.54	0.88	0.114	0.505	0.792
Lauric aldehyde	0.22	0.25	0.34	0.34	0.024	0.022	0.070
1-Octanal	2.34 ^a^	1.67 ^b^	2.01 ^ab^	1.58 ^b^	0.093	0.160	0.004
1-Nonanal	4.17	3.06	4.57	2.26	0.499	0.798	0.606
5-Methoxy-3,4-methylenedioxybenzaldehyde	3.11	2.99	5.73	2.57	0.496	0.097	0.143
γ-Nonalactone	0.18	0.16	0.13	−	0.006	−	−
Hexanal	2.12	2.12	2.59	1.89	0.111	0.103	0.177
Ketones							
Methylisohexenyl ketone	0.58	0.44	0.40	0.35	0.042	0.060	0.149
2-Octanone	0.30 ^b^	0.23 ^bc^	0.15 ^c^	0.45 ^a^	0.031	0181	0.001
Hydrocarbon							
Methylbenzene	0.67	0.68	0.51	0.67	0.049	0.693	0.702
Ethylbenzene	0.19	0.20	−	0.17	0.011	−	−
n-Octane	0.33 ^a^	0.13 ^c^	0.18 ^b^	0.17 ^b^	0.0017	0.001	<0.001
n-Heptadecane	0.88	−	0.99	0.68	0.046	−	−
n-Nonadecane	0.83 ^a^	0.28 ^b^	0.26 ^b^	0.94 ^a^	0.079	0.669	<0.001
Alcohols							
Hexanol	0.66 ^b^	0.83 ^ab^	0.67 ^b^	1.02 ^a^	0.044	0.161	0.020
Heptanol	2.83 ^ab^	3.39 ^a^	2.26 ^b^	2.92 ^ab^	0.141	0.317	0.032
octanol	8.54	8.58	8.27	8.73	0.477	0.915	0.948
1-octen-3-ol	4.96 ^b^	5.37 ^b^	4.92 ^b^	9.60 ^a^	0.706	0.038	0.035
Trans-2-octen-1-ol	−	0.23	0.24	1.29	0.175	−	−
Butyl carbinol	0.53 ^b^	0.88 ^b^	0.75 ^b^	1.51 ^a^	0.102	0.031	0.013
3,7,11-trimethyl-1-ol	0.78	0.30	0.33	0.33	0.067	0.618	0.865
Esters							
Isopropyl myristate	0.46	0.67	0.17	0.30	0.061	0.068	0.189
Octano-1,4-lactone	0.17	0.13	−	−	0.007	−	−
Methyl N,N-diethylcarbamodithioate	8.56	10.25	7.66	6.25	0.803	0.191	0.270
Other categories							
Pentylfuran	0.73	0.64	0.92	0.88	0.081	0.342	0.637
Carbon disulfide	1.08	0.82	1.06	1.02	0.077	0.963	0.787
Trimethylamine	0.22	0.21	0.18	0.19	0.008	0.096	0.217
Formamide, N,N-dibutyl	17.88	18.91	16.06	14.11	1.483	0.296	0.520

Note: “−” indicates not detected. Different letters (a, b, c) marked on the same row of data indicate significant differences (*p*< 0.05).

**Table 5:** ROAV of volatile flavor compounds in *Longissimus lumborum* of yak.

Index	ROAV				Perception Threshold (μg/kg)
	CON	RPM5	RPM10	RPM15	
Aldehyde					
Benzaldehyde	0.60	0.70	0.51	0.29	750.89
Hptanal	11.87	14.78	13.21	4.89	2.8
Lauric aldehyde	42.00	54.45	62.09	40.56	0.13
1-Octanal	100.00	78.83	82.08	41.84	0.59
1-Nonanal	95.82	77.74	100.00	32.08	1.1
5-Methoxy-3,4-methylenedioxybenzaldehyde	0.71	0.76	1.25	0.37	110
γ-Nonalactone	0.47	0.45	0.32	−	9.7
Hexanal	10.69	11.86	12.43	5.92	5
Ketones					
Methylisohexenyl ketone	0.21	0.18	0.14	0.08	68
2-Octanone	0.15	0.13	0.07	0.14	50.2
Hydrocarbon					
Methylbenzene	0.03	0.04	0.02	0.02	524
Alcohols					
Hexanol	2.98	4.15	2.89	2.85	5.6
Heptanol	13.24	17.52	10.07	8.45	5.4
Octanol	1.71	1.90	1.58	1.08	125.8
1-octen-3-ol	83.53	100.00	78.89	100.00	1.5
Trans-2-octen-1-ol	−	0.32	0.29	1.01	20
Butyl carbinol	0.09	0.16	0.12	0.16	150.2
Others					
Octano-1,4-lactone	0.67	0.56	−	−	6.5
Pentylfuran	3.19	3.10	3.81	2.36	5.8

Note: “−” denotes not detected.

## Data Availability

The original contributions presented in this study are included in the article/Supplementary Materials. Further inquiries can be directed to the corresponding author. The raw sequencing data generated in this study have been deposited in the NCBI Sequence Read Archive (SRA) under BioProject accession number PRJNA1229162.

## Supplementary Materials

The following supporting information can be downloaded at https://www.mdpi.com/article/10.3390/foods14122102/s1, Table S1. primers sequence of differential genes.
