# Supplementary material for: Effects of Rumen-Protected Methionine on Meat Quality, Fatty Acid Composition, Volatile Flavor Compounds and Transcriptomics of Longissimus lumborum of Yak (Bos grunniens)
Source: Foods. 2025 Jun 15;14(12):2102. doi: 10.3390/foods14122102 (PMC12191923; doi:10.3390/foods14122102)
Supplement: Supplementary file 1 [file foods-14-02102-s001.zip › Supplementary File.pdf]

Table S1. primers sequence of differential genes

Gene	Primer sequence (5'→3')
GAPDH	F:CCACGAGAAGTATAACAACACC
	R: GTCATAAGTCCCTCCACGAT
RPL27A	F: CTGCCCAACTGTCAACCTTG
	R: AGTAACCTGATCGCACCACA
CTGF	F: GAATGGGTGTGTGATGAGCC
	R: CTCGGATCATGGTTGGGTCT
RPL8	F: AGTCATCTCCCACAACCCTG
	R: GATGCTCGACAGGGTTCATG
RPLP2	F: TGCGTTACGTTGCCTCATAC
	R: CTGATGACCTTGTTGAGCCG
RPL36A	F: TTCTGTGCTGGTATCGCTCT
	R: AGCCACTCTGTTTCCTGTCA
S100A1	F: GGGCAAAGAGGGAGACAAGT
	R: CCGTTCTCATCCAGCTCCTT
